# Acute Glycemic and Hemodynamic Responses to Single- and Multi-Joint Resistance Exercises in Individuals with Type 2 Diabetes: A Pilot Randomized Crossover Study

**DOI:** 10.3390/ijerph22081288

**Published:** 2025-08-18

**Authors:** Rodrigo Sudatti Delevatti, Fábio Duarte da Silva, Filipe De Lucca Braga, Lucineia Orsolin Pfeifer, Maria Eduarda de Moraes Sirydakis

**Affiliations:** 1 Grupo de Pesquisa em Exercício Clínico (GPEC), Centro de Desportos, Universidade Federal de Santa Catarina, R. Deputado Antônio Edu Vieira, Pantanal, Florianópolis 88040-001, Brazil; fabio.duarte@grad.ufsc.br (F.D.d.S.);; 2 Faculdade de Motricidade Humana, Universidade de Lisboa, Campo Grande, 1749-016 Lisboa, Portugal; filipebraga@edu.ulisboa.pt; 3 Hospital de Clínicas de Porto Alegre, Universidade Federal do Rio Grande do Sul, Pantanal, Florianópolis 88040-001, Brazil; lorsolinpfeifer@gmail.com

**Keywords:** physical exercise, strength training, diabetes, hypertension, glycemia

## Abstract

Introduction: There is a lack of knowledge regarding the acute glycemic and blood pressure responses to resistance exercises that involve different amounts of muscle mass. Objective: To analyze the acute effects of single- and multi-joint resistance exercises on glycemic control and blood pressure in individuals with type 2 diabetes (T2DM). Methods: This is a pilot randomized crossover trial, including adults with T2DM of both genders. The participants performed three sessions (two experimental sessions: one with single-joint exercises (SIN) and the other with multi-joint exercises (MULTI); and a control session (CON)) in a randomized order, with outcomes being evaluated pre-exercise, immediately, 15 and 30 min after the sessions. Both sessions consisted of five exercises performed in three sets of 10 to 12 maximum repetitions. Analyses were performed by generalized estimation equations. Results: Fifteen adults (including eleven women) participated in this study. Both experimental sessions showed glycemic reductions immediately after the sessions (MULTI: −17 mg/dL; SIN: −29 mg/dL; *p* < 0.001), and these values were kept similar up to 30 min after the session. The control session presented a glycemic reduction immediately after the session (−18 mg/dL), which increased 15 min later (−29 mg/dL), stabilizing up to 30 min after the session. Systolic blood pressure was increased immediately after both experimental sessions, retuning to baseline values 15 min post-session. Diastolic blood pressure increased in the control session with time, without any alterations in the experimental sessions. Conclusions: Similar glycemic reductions were found in the experimental sessions, without superiority over the control session. Minimal changes were found in blood pressure.

## 1. Introduction

The prevalence of diabetes worldwide is alarmingly high and continues to rise rapidly, with type 2 diabetes (T2D) accounting for approximately 90% of cases [1]. This condition is strongly associated with various cardiovascular diseases, particularly hypertension, which significantly increases cardiovascular morbidity and mortality [2]. Approximately 50% of individuals with T2D also have hypertension, making the management of both conditions essential for improving quality of life and reducing the risk of cardiocerebrovascular events [3].

Alongside diet and medication, exercise is widely recognized as a fundamental component in the management of T2D [4,5]. Both aerobic and resistance exercises are recommended as effective strategies [4,5,6]. While chronic exercise adaptations provide the most clinically significant benefits, understanding the acute effects of exercise is crucial for optimal training prescription. Only by assessing the immediate clinical responses to each session can safe and effective exercise recommendations be made.

A recent meta-analysis [7] demonstrated that continuous aerobic exercise reduced blood glucose levels by 26.7 mg/dL, while interval aerobic exercise resulted in a more substantial reduction of 47.92 mg/dL one minute after exercise, with effects persisting for up to 30 min post-session. In contrast, resistance exercise led to a reduction of 21.26 mg/dL one minute after the session; however, this effect was not sustained beyond 10 min.

The acute glycemic effects of aerobic exercise have been widely studied, with its safety and efficacy well established in individuals with T2D. However, less is known about the acute effects of resistance exercise, particularly regarding training variables that may influence clinical responses, such as exercise selection. Multi-joint exercises, which engage larger muscle groups, may have a greater impact on metabolic and cardiovascular outcomes compared to single-joint exercises. While single-joint exercises are easier to perform and require less technical skill, multi-joint exercises may elicit superior functional and glycemic benefits despite their increased complexity [8,9]. Nonetheless, this hypothesis remains unclear.

Regarding acute blood pressure responses, Mello et al. (2016) [10] found that multi-joint resistance exercises induced lower cardiovascular overload, measured by the double product, and greater post-exercise hypotensive effects compared to single-joint resistance exercises in trained young men.

Although major exercise guidelines for T2D [4,5] recommend resistance training involving large muscle groups, further investigation is needed to clarify the acute glycemic and blood pressure responses to single- and multi-joint resistance exercises, particularly through randomized controlled crossover trials. Thus, the aim of the present study is to analyze the acute glycemic and blood pressure responses to single- and multi-joint resistance exercise sessions in individuals with T2D. We hypothesize that the effects of the multi-joint session will be more pronounced.

## 2. Methods

### 2.1. Design

This study is a pilot randomized crossover trial involving individuals with T2D who participated in three sessions: two experimental sessions—one comprising single-joint resistance exercises and the other comprising multi-joint resistance exercises—and a control session without exercise. The trial is registered in the Brazilian Clinical Trials Registry System (code RBR-102jnmyx; https://www.backuptrials.com/bt/default/show_trial?trial_id=RBR-102jnmyx; (accessed on 24 June 2025)).

### 2.2. Participants

Individuals with T2D of both sexes, aged 18 years or older, were included in this study. Participants met the following inclusion criteria: a diagnosis of T2D confirmed by a blood test or the use of antidiabetic medication, ongoing endocrinological treatment, and a pre-exercise glycemic range between 90 and 250 mg/dL to ensure safe exercise participation [11]. The exclusion criteria for this study included the presence of severe autonomic neuropathy, severe peripheral neuropathy or a history of foot injuries, proliferative diabetic retinopathy, severe non-proliferative diabetic retinopathy, decompensated heart failure, peripheral amputations, musculoskeletal impairments preventing the execution of resistance exercises, and the inability to travel for study visits. The eligibility was initially evaluated in the first contact, through phone calls or WhatsApp, and confirmed after face-to-face anamnesis containing the clinical history and checklist of eligibility. Recruitment was conducted by social media advertisements of the Grupo de Pesquisa em Exercício Clínico (GPEC) and researchers involved in this study. Additionally, a list of patients with T2D obtained from an outpatient clinic was utilized, and individuals who had previously expressed interest in research participation were contacted by a messaging application, where they received detailed information about the study procedures.

### 2.3. Randomization and Blinding

The order of the training sessions was randomized using the website www.randomizer.org (accessed on 24 June 2025) in a counterbalanced manner by a researcher not directly involved in the experimental procedures. Due to the nature of the study, blinding of the evaluators was not possible, as they were responsible for administering the intervention and collecting data in each session. Our randomization used blocks of 3 participants, applying three possible sequences until reaching the total sample of 15 participants. This approach ensured a balanced distribution, with an identical number of participants starting in each of the three session types, thereby minimizing any potential order effects.

### 2.4. Experimental Procedures

The experimental procedures consisted of five visits. During the first visit, participants were informed about the research procedures, benefits, and potential risks. They had the opportunity to clarify any questions before signing the Free and Informed Consent Form. Following this, an anamnesis form was administered to collect data for sample characterization, including sociodemographic information, health status, physical activity habits, and medication use. Subsequently, blood pressure, blood glucose levels, and anthropometric measurements (height, body mass, and waist circumference) were assessed. During the second visit, participants were familiarized with the exercise sessions, and the external load for each exercise was determined. The third, fourth, and fifth visits involved the randomized implementation of the experimental and control sessions, with a minimum interval of 48 h between sessions. In the experimental sessions, blood glucose and blood pressure were measured before and after the intervention (immediately, as well as 15 and 30 min post-exercise). At the end of each exercise session, the rate of perceived exertion (RPE) and affective valence were recorded. For these sessions, individuals had to be fed, without any special recommendations, and no food intake was carried out during the procedures.

### 2.5. Interventions

Each exercise session lasted approximately 35 min and was divided into a joint warm-up (five minutes) and a main session (approximately 30 min). The main session consisted of five resistance exercises, with one session focused on single-joint exercises and another on multi-joint exercises, performed on different days. The intervention team consisted of three physical education professionals with prior training in working with clinical populations. The structure of the exercise sessions is presented in Table 1.

### 2.6. Control Session

In the control session, participants remained at rest for 10 min, after which their blood pressure and glycemia were assessed. They stayed in the gym for the same duration as the exercise sessions, moving around the weight training equipment without engaging in any exercises. Following this period, participants underwent the same data collection procedures as in the experimental sessions. The main part of the control session consisted of passive activity (sedentary behavior) performed in five different areas of the gym. Participants remained seated for 6 min at each station (weight machines), walking at a low intensity to transition between them. This approach was adopted to simulate participants’ daily movements and ensure similarity to the experimental sessions, except for the prescribed exercises.

### 2.7. Anthropometric Measurements

Anthropometric assessments were conducted to characterize the study sample. Body mass was measured using a Sohenle^®^ (Backnang, Germany) scale with a resolution of 50 g, with participants wearing appropriate clothing for the measurement. Height was determined using a Sanny^®^ (São Bernardo do Campo, Brazil) stadiometer, with individuals standing barefoot. Body mass index (BMI) was calculated based on these measurements. Waist circumference was assessed using a non-elastic measuring tape positioned at the midpoint between the anterior superior iliac crest and the last rib.

### 2.8. Internal Load

The internal load of each exercise session was quantified using the rate of perceived exertion (RPE) scale adapted from Borg [12]. This assessment aimed to estimate exercise intensity after each session, and participants were individually asked to report their perceived exertion at the end of each session.

### 2.9. External Load

The external load was determined based on the weight used for each exercise, which was established during the familiarization phase, considering the individual capabilities of each participant.

### 2.10. Affective Response

Affective valence was assessed using a validated scale to evaluate participants’ subjective experience of pleasure or displeasure in response to physical exercise. The scale [13] ranges from −5 to +5, where −5 represents “very bad” and +5 represents “very good.” Intermediate descriptors include the following: −3 = bad; −1 = reasonably bad; 0 = neutral; +1 = reasonably good; and +3 = good.

### 2.11. Capillary Glycemia—Primary Outcome

Capillary glycemia was measured by a trained research team member using an Accu-Check glucometer (Performa model, Roche, Portugal). Only participants with pre-exercise glycemia levels between 90 and 250 mg/dL were permitted to begin the training sessions.

### 2.12. Blood Pressure Assessment

Blood pressure was measured by a trained professional using an OMRON device (model HEM 7122) with appropriately sized cuffs based on participants’ arm circumferences, following the Brazilian Hypertension Guidelines [14]. Participants remained seated in a calm environment for five minutes before the measurement. Only individuals with systolic and diastolic blood pressure values below 160 mmHg and 105 mmHg, respectively, were allowed to commence the exercise sessions.

### 2.13. Adverse Events’ Monitoring

The research team was trained to observe, interview, and document any adverse events occurring during the sessions.

### 2.14. Ethical Considerations

This study was approved by the Ethics Committee for Research Involving Human Participants at UFSC (CEPSH/UFSC) under protocol number 5.486.869. All participants were informed about the research procedures, potential risks, and benefits, and they were explicitly advised of their right to withdraw from the study at any time.

### 2.15. Data Analysis

The sample size calculation was based on unpublished data from the GPEC, estimating a mean difference of 9 mg/dL (effect size f: 0.215) in glycemic reduction favoring the multi-joint exercise session. The calculation was performed using the G*POWER 3.1 software, assuming a significance level of 0.05, a statistical power of 80%, and a correlation coefficient of 0.5. The resulting sample size was 14 individuals. Descriptive data are presented as mean and standard deviation, while inferential results are reported as mean and standard error. Differences between pre- and post-intervention measurements and between sessions (time effect, session effect, and time*session interaction effect) were analyzed using the generalized estimating equation (GEE) method. Multiple comparisons were conducted using the LSD post hoc test. For nonparametric data, the Wilcoxon test was applied. No model adjustment (e.g., for baseline glucose levels or other confounders) was used in the analysis. The significance level was set at 5%, and all analyses were performed using the Statistical Package for the Social Sciences (SPSS), version 23.0.

## 3. Results

**Figure 1:**
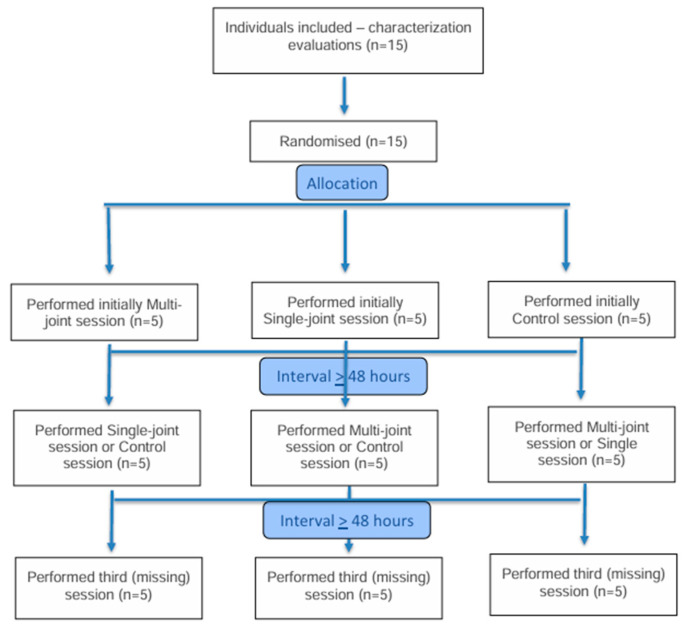
Flow diagram of the participants in the three sessions.

The flow diagram of the participants in the three sessions is presented in Figure 1. The main characteristics of the participants are present in Table 2. The major part of the sample was middle-aged women, who were overweight and had hypertension.

**Figure 2:**
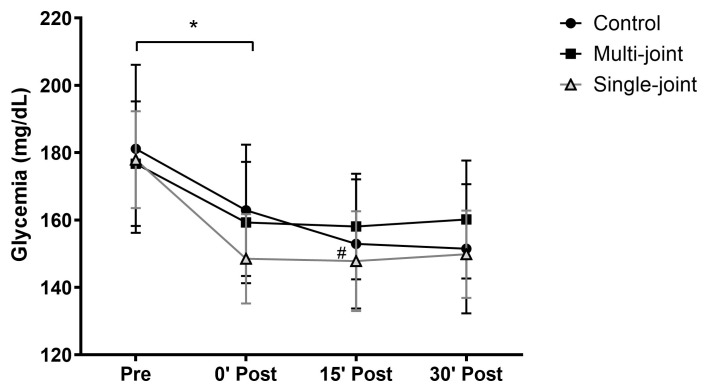
Glycemic levels Pre and 0 (0′ Post), 15 (15′ Post), and 30 (30′ Post) minutes after control (circle), multi-joint (square), and single-joint (triangle) sessions. * Difference between Pre and 0′ Post for all sessions; # 15′ Post different from 0′ Post only for control session. Values are presented as mean and standard error.

Regarding glycemia, only an isolated time effect (*p* < 0.001) was found (Figure 2). Both experimental sessions presented significant glycemic reductions immediately after the sessions (multi-joint: −17 mg/dL; single-joint: −29 mg/dL), with these values remaining similar up to 30 min after the sessions. The control session presented a glycemic reduction immediately after exercise (−18 mg/dL), which increased 15 min later (−29 mg/dL), stabilizing up to 30 min after the session. It is important to highlight that these reductions occurred from very similar baseline glycemic values (control: 181 ± 25 mg/dL; multi-joint: 177 ± 18 mg/dL; single-joint: 178 ± 14 mg/dL).

Table 3 and Table 4 present the results of blood pressure. Regarding systolic blood pressure, both exercise sessions increased its values immediately after the session, returning to its baseline values 15 min after sessions, and remaining similar until 30 min after the sessions. Interestingly, only the multi-joint session presented higher values immediately after the session than those observed in the control session at the same time point.

Regarding diastolic blood pressure, in the multi-joint session, the values 15 min post-session were higher than the values immediately post-session. In the single-joint session, no changes were found with time; however, its 0′ post-session values were superior to 0′ post-session values of the multi-joint session. For the control session, the 15 and 30 min post-session values were higher than the pre-session values, and the 30′ post-session value was also higher than the multi-joint session value at the same time point.

Comparing the 30 min post-session values with the pre-session values, mean differences for glycemia of −17 mg/dL, −29 mg/dL, and −30 mg/dL were found in the multi-joint, single-joint, and control sessions, respectively. For SBP, mean differences of −2.33 mmHg, −1.47 mmHg, and 2.73 mmHg were found in the multi-joint, single-joint, and control sessions, respectively, and for DBP, mean differences of 1.40 mmHg, 0.27 mmHg, and 3.47 mmHg were found in the multi-joint, single-joint, and control sessions, respectively.

The exercise sessions presented similar RPE session and affectivity (Table 5). The median values of the RPE session corresponded to few strong and moderate intensities for multi-joint and single-joint sessions, respectively. For affectivity, both sessions presented very good median values.

No adverse events related to exercise occurred in any session. When participants arrived with glycemic or blood pressure values above the permitted range for physical activity, the intervention team provided appropriate assistance and waited for the values to normalize before proceeding or rescheduled the session if necessary.

## 4. Discussion

In general, glycemic levels decreased similarly across all sessions. Regarding systolic blood pressure, only the exercise sessions showed the expected immediate post-exercise increase, resulting in higher values compared to the control session at the same time point. For diastolic blood pressure, although some fluctuations were observed over time, the main finding was a lower diastolic blood pressure value in the multi-joint session compared to the control session (−4.47 mmHg), indicating the presence of post-exercise hypotension (PEH).

Regarding glycemic response, this study refutes the hypothesis that multi-joint exercises lead to superior glycemic reduction compared to single-joint exercises. Additionally, no differences were found between the experimental and control sessions. From a practical perspective, this finding is relevant, as the Diabetes Society Guidelines [4,5] recommend resistance training with two to three sets for large muscle groups, suggesting that both exercise types may provide acute glycemic benefits. Given the limitations that many individuals with T2D face when starting more complex exercise programs requiring greater motor coordination, single-joint exercises may serve as a viable alternative. However, despite these practical considerations, the lack of difference in glycemic response between control and exercise sessions suggests that the observed reductions may not be solely attributable to the exercises performed.

Although pre-session glycemic values were similar across conditions, the lack of dietary control before sessions may have influenced glycemic behavior, potentially explaining reductions observed in the control session. Similarly, Ferrari et al. (2021) [15] reported no differences in glycemic response between bodyweight resistance training and a control session in middle-aged adults with hypertension.

The glycemic reduction found in our study (between 17 and 29 mg/dL immediately after exercise) is similar to the reduction observed by de Almeida et al. (2025) [7] one minute after resistance exercise sessions (21 mg/dL). However, in that study, the glycemic response was not maintained throughout the 30 min post-session, in contrast to the sustained glycemic reduction observed in our study.

For systolic blood pressure, the immediate post-exercise increase was expected due to increased cardiac output but remained within safe limits and subsequently decreased over time. The absence of post-exercise hypotension (PEH) could be attributed to the relatively low pre-exercise values (~125 mmHg) and the short post-exercise monitoring period (30 min). Ferrari et al. (2021) [15] reported lower systolic blood pressure values compared to a control session only 45 min post-exercise, while Silva et al. (2020) [16] found significant differences in diastolic blood pressure 30 min post-session. In the present study, despite the lack of significant differences between control and exercise sessions, systolic blood pressure in the control condition showed an increasing trend at 30 min post-session, with mean differences of 5.53 mmHg and 5.00 mmHg compared to the multi-joint and single-joint sessions, respectively—both of which exhibited a decreasing trend.

For diastolic blood pressure, there was no immediate increase post-exercise, indicating cardiovascular safety. However, in the control session, despite participants moving between equipment to minimize prolonged sedentary behavior, diastolic blood pressure increased at 15 and 30 min post-session. This led to a significant difference between the multi-joint and control sessions, a notable finding in line with previous studies [15,16] using multi-joint resistance exercises. The absence of a significant reduction in diastolic blood pressure from pre- to post-exercise is likely due to the already low baseline values (70–74 mmHg), leaving little room for further reduction [17].

Regarding internal load, measured by RPE session, both exercise sessions were classified as moderate to somewhat difficult, aligning with the recommended intensity levels. These intensities appear safe and potentially effective, particularly in the initial stages of training programs. However, for a greater cardiometabolic impact from resistance training alone, a higher training volume (e.g., additional exercises) may be necessary.

Another secondary variable assessed was affectivity, with both sessions yielding a median rating of 5 (“very good”), suggesting that participants enjoyed both exercise modalities. This is an important factor, as enjoyment of exercise can enhance adherence to regular physical activity, particularly in clinical populations [18].

One notable finding is that even simple, single-joint exercises demonstrated moderate intensity, very good affective responses, and short-term glycemic benefits for individuals who may be unable to perform more complex movements, appearing to be an interesting alternative.

### Limitations and Strengths

The limitations of this study include the lack of dietary control before sessions, the short post-session monitoring period, and the lower sample size, limiting the external validity of the findings. The other limitation is not having evaluated important mechanisms involved in the relation between exercise and cardiometabolic outcomes, such as inflammatory markers, for better understanding the results. However, notable strengths include the complementary evaluation of RPE session and affectivity and the controlled crossover design with a randomized session order.

## 5. Conclusions

Both experimental and control sessions resulted in glycemic reductions, with no evidence of exercise-induced superiority or differences between the two exercise modalities. Regarding blood pressure, PEH was observed only for diastolic blood pressure at 30 min post-session in the multi-joint session, with values lower than those in the control session. Studies evaluating glycemic and blood pressure responses over longer post-exercise periods (e.g., 24 h), under controlled dietary conditions, are important for a better understanding of the cardiometabolic effects of resistance training sessions involving different amounts of muscle mass.

## Figures and Tables

**Table 1:** Structure of the exercise sessions.

Single-Joint Exercises	Multi-Joint Exercises	Volume and Intensity
Chest fly	Bench press	Three sets to 10–12 RM Rest interval: 60–90 s
Knee extension	Leg press horizontal	
Reverse chest fly	Low row	
Knee flexion	Leg press 45°	
Side elevation–shoulder	Shoulder press	

RM: repetition maximum.

**Table 2:** Participants characteristics (n: 15).

Variables	Values
Age (years)	61.93 ± 9.99
Stature (cm)	1.70 ± 0.09
Body mass (kg)	78.79 ± 13.36
BMI (kg/m^2^)	29.20 ± 4.60
Hypertension	11 (73.3%)
**Medicines**	
Hypoglycemic	15 (100%)
Antihypertensives	11 (73.3%)
Antilipemic	4 (26.7%)
Antidepressants	2 (13.3%)
**Marital Status**	
Married	11 (73.3%)
Divorced	1 (6.7%)
Single	3 (20%)
**Education level**	
Elementary school complete	2 (13.3%)
Elementary school incomplete	2 (13.3%)
High school complete	4 (26.7%)
High school incomplete	2 (13.3%)
Higher education complete	4 (26.7%)
Higher education incomplete	1 (6.7%)
**Physical exercise**	
Yes	8 (53.3%)
No	7 (46.7%)
**Modality**	
Walking	4 (50%)
Gymnastic	2 (25%)
Weight training	1 (12.5%)
Cycling	1 (12.5%)
Pilates	2 (25%)
Combined training	1 (12.5%)
Running	1 (12.5%)
**Exercise frequency**	
One to two times a week	5 (62.5%)
Three to four times a week	3 (37.5%)

Data described by mean and standard deviation or absolute and relative frequency. BMI = body mass index.

**Table 3:** Systolic blood pressure behavior in two experimental sessions and one control session.

Moments	Multi-Joint Session	Single-Joint Session	Control Session
SBP pre-session	125.00 ± 3.62	124.67 ± 3.59	125.47 ± 2.87
SBP 0′ post-session	141.00 ± 5.27 *^a^	131.67 ± 4.18 *^a^	130.60 ± 4.58 ^b^
SBP 15′ post-session	124.93 ± 4.22	122.93 ± 4.06	126.33 ± 4.24
SBP 30′ min post-session	122.67 ± 4.52	123.20 ± 3.40	128.20 ± 3.83

SBP: systolic blood pressure. * Different from other moments of evaluation. Different letters indicate significant differences among sessions at the same time point. Values are presented as mean and standard error.

**Table 4:** Diastolic blood pressure behavior in two experimental sessions and one control session.

Moments	Multi-Joint Session	Single-Joint Session	Control Session
DBP Pre-session	70.80 ± 2.87	73.80 ± 2.18	73.20 ± 2.09
DBP 0′ post-session	70.33 ± 2.17 ^a^	74.20 ± 2.11 ^b^	74.53 ± 2.77 ^ab^
DBP 15′ post-session	73.07 ± 2.59 ^#^	74.20 ± 2.13	75.73 ± 2.09 ^£^
DBP 30′ min post-session	72.20 ± 2.52 ^a^	74.07 ± 2.23 ^ab^	76.67 ± 2.59 ^£b^

DBP: diastolic blood pressure. # Different from the 0′ post-session. £ Different from the baseline values. Different letters indicate significant differences among sessions at the same time point. Values are presented as mean and standard error.

**Table 5:** RPE session and affectivity in multi-joint and single-joint sessions.

	Multi-Joint Session	Single-Joint Session	*p*-Value
RPE session	4 (3–4)	3 (3–4)	0.891
Affectivity	5.0 (4.5–5.0)	5.0 (3.0–5.0)	>0.999

RPE: rate of perceived exertion. Data are presented as median and interquartile range.

## Data Availability

The original contributions presented in this study are included in the article. Further inquiries can be directed to the corresponding author.
